# Editorial: Human decision-making in combat situations involving traditional and immersive visual technologies

**DOI:** 10.3389/fpsyg.2023.1166115

**Published:** 2023-05-17

**Authors:** Varun Dutt, Sushil Chandra

**Affiliations:** ^1^Applied Cognitive Science Laboratory (ACS Lab), Indian Institute of Technology Mandi, Mandi, India; ^2^Department of Biomedical Engineering, Defence Research and Development Organisation (DRDO), New Delhi, India

**Keywords:** human decision-making, immersive visual technologies, combat situation, decision aids, defense

Traditional and immersive visual technologies (such as virtual reality and augmented reality) can potentially be used to enhance decision-making in combat situations by providing a more immersive and interactive training environment for users. These interfaces can assist people to practice and simulate various scenarios, such as combat situations, in a controlled environment, which can assist them in developing and improving their decision-making skills and cognitive performance. Besides, immersive visual interfaces can provide a more realistic representation of the situation, which can aid in developing situational awareness and tactical planning abilities. However, the effectiveness of these interfaces in enhancing decision-making among relevant stakeholders will depend on various factors, such as the design of the training program and the quality of the virtual environment. This Research Topic focuses on new insights, novel developments, current challenges, latest discoveries, recent advances, and future perspectives in the applications of traditional and immersive technologies in enhancing human decision-making in complex situations and simulations, with a particular focus on combat situations. Therefore, the main goal of this special Research Topic is to shed light on the progress made in the recent past in the field and on its future challenges to provide a thorough overview.

This Research Topic brings together several contemporary research articles of relevance. For example, moving from individuals to teams, Ouverson et al. evaluate the performance and team skills of teams working with an Intelligent Team Tutoring System on a virtual military surveillance task. Higher levels of role and task experiences show significant and medium-sized effects on communication performance. Similarly, another original research (de Melo et al.) evaluates the efficacy of a self-embodied assistant designed to assist military personnel simultaneously and to minimize the user's cognitive load. Participants are paired with a voice assistant, an embodied assistant, or no assistant. Results indicate that the embodied assistant achieves higher performance with a smaller cognitive burden on the decision-maker than the voice assistant and no assistant. Hebbar et al. derive a non-invasive technique to estimate a pilot's cognitive workload. To estimate cognitive workload, these researchers utilize pilots' physiological indications such as electroencephalographic (EEG) signals, ocular parameters, and pilot performance-based quantitative metrics. Results indicate that the EEG's beta frequency band, nearest neighborhood index specifying the distribution of gaze fixation, L1 Norm of power spectral density of pupil diameter, and the duty cycle metric indicated variations in cognitive workload during flying. In addition, another original research article (Rao et al.) evaluates the efficacy of different repetitive training conditions in immersive virtual reality on dynamic decision-making in a complex search-and-shoot environment. Results reveal that participants in the heterogenous and difficult training conditions performed significantly better after the intervention compared to before the intervention.

Kase et al. provide a perspective on three areas of development for future warfighter-machine interfaces: AI-directed decisional guidance, computationally informed decision-making, and realistic representations of decision spaces. According to these authors, the next-generation wargaming platforms can reduce risk, decrease operating costs, and improve overall outcomes for increasingly complex military operating environments.

Overall, this Research Topic focuses on developing traditional and immersive technologies for strategic decision-making. It is hoped that this Research Topic may help to inform and provide direction to researchers at the forefront of developing traditional and immersive visual technologies for understanding and improving human decisions.

## Author contributions

VD supervised and wrote the editorial article. SC reviewed the final version of the editorial article. Both authors approved the final version of the manuscript for publication.

